# Synthesis and role of melanin for tolerating *in vitro* rumen digestion in *Duddingtonia flagrans*, a nematode-trapping fungus

**DOI:** 10.1080/21501203.2019.1631896

**Published:** 2019-06-20

**Authors:** Deivid França Freitas, Olney Vieira-Da-Motta, Luciana Da Silva Mathias, Roberto Weider De Assis Franco, Raphael Dos Santos Gomes, Ricardo Augusto Mendonça Vieira, Letícia Oliveira Da Rocha, Fabio Lopes Olivares, Clóvis De Paula Santos

**Affiliations:** a Laboratório de Biologia Celular e Tecidual, Centro de Biociências e Biotecnologia, Universidade Estadual do Norte Fluminense Darcy Ribeiro, Campos dos Goytacazes, RJ, Brazil; b Laboratório de Sanidade Animal, Centro de Ciências e Tecnologias Agropecuárias, Universidade Estadual do Norte Fluminense Darcy Ribeiro, Campos dos Goytacazes, RJ, Brazil; c Laboratório de Ciências Físicas, Centro de Ciências Tecnológicas, Universidade Estadual do Norte Fluminense Darcy Ribeiro, Campos dos Goytacazes, RJ, Brazil; d Laboratório de Zootecnia, Centro de Ciências e Tecnologias Agropecuárias, Universidade Estadual do Norte Fluminense Darcy Ribeiro, Campos dos Goytacazes, RJ, Brazil

**Keywords:** Duddingtonia flagrans, nematode-trapping fungi, melanin, L-DOPA, tricyclazole

## Abstract

We describe the synthesis and a function of melanin in *Duddingtonia flagrans*, a nematode-trapping fungus. We tested various culture media treated with L-DOPA, glucose and tricyclazole on fungal growth and melanin distribution using infrared spectroscopy (IS), electron paramagnetic resonance (EPR) and transmission electron microscopy (TEM). *In vitro* rumen digestion was used to test the environmental stress and then to evaluate the capacity of this fungus to trap nematode larvae. The growth and melanization of the fungus after 21 days of incubation at 30°C were best in Sabouraud dextrose medium. IS indicated the presence of melanin in *D. flagrans*, with similar bands for commercial melanin used as a control, and assigned the values obtained by EPR (g of 2.0051 ± 0.0001) to the production of melanin by the fungus. TEM indicated that melanin was produced in melanosomes but was not totally inhibited by tricyclazole. Within the limits of experimental error, the predatory activity of fungus treated with tricyclazole was drastically affected after 27 h of *in vitro* anaerobic stress with rumen inoculum. The deposition of melanin particles on the fungal cell wall contributed to the maintenance of *D. flagrans* predatory abilities after *in vitro* anaerobic ruminal stress.

## Introduction

Melanins are amorphous substances formed by the polymerization of phenolic and indolic compounds and are found in all biological systems, indicating an early evolutionary origin (Butler et al. 2009; Solano 2014). Melanin in the cell walls of fungi can increase survival by protecting against ultraviolet radiation (Allam and El-Zaher 2014), traumatic damage (Eisenman and Casadevall 2012) and extreme temperatures and pressures in, for example, polar regions or deserts (Rosa et al. 2010). Melanin-bearing fungi can survive inside domestic machines, e.g. dishwashers, where they apparently resist the heat and action of detergents (Zalar et al. 2011), and even in inhospitable environments, such as in radiation-contaminated nuclear reactors (Zhdanova et al. 2000; Dadachova et al. 2008). Melanin can also increase the virulence and pathogenicity of many fungal species (Zhdanova et al. 2000; Tian et al. 2003; Plonka and Grabacka 2006; Casadevall et al. 2012).

Melanin pigments are very common in the Fungi kingdom (Solano 2014), although melanogenesis is restricted to certain stages of mycelial development and sporulation or to defensive reactions against damage by environmental stressors. They are thus abundant and can be associated with cell walls or subcellular organelles (Franzen et al. 2008). Melanin-producing organisms can usually synthesize it by two main pathways, either from an endogenous substrate involving 1,8-dihydroxynaphthalene (DHN) and the action of polyketide synthase, or alternatively by an exogenous pathway with the addition of L-3,4-dihydroxyphenylalanine (L-DOPA) (Almeida-Paes et al. 2009). The DHN and L-DOPA melanin precursors are segregated, oxidized and externalized in the cell wall of melanized fungi and yeasts (MHG et al. 2014). Despite the importance and ubiquity of this pigment, the details of its chemical structure are not well understood, because melanins are not easily soluble in organic solvents and are structurally complex molecules, diverse and still not well defined (Eisenman and Casadevall 2012). Conventional biochemical techniques thus cannot often be applied.

Among the diversity of fungi in nature, nematode-trapping fungi have the ability to capture and infect nematodes are thus of interest due to their potential for biocontrol (Larsen 2006). *Monacrosporium haptotylum* and *Arthrobotrys oligospora*, both nematophagous fungi, produce tyrosinase (Meerupati et al. 2013), which is one of the most highly upregulated proteins used in a capture structure compared to mycelium proteins in *M. haptotylum* (Andersson et al. 2013). Fungal tyrosinases are cytosolic enzymes involved in melanogenesis that are more heterogeneous than other enzymes (Bell and Wheeler 1986). Tyrosinases have a range of molecular weights and are generally associated with the formation and stability of spores in defensive mechanisms and increased virulence, tissue regeneration in cases of traumatic damage and a fundamental role in pigmentation for some fungal species (Halaouli et al. 2006).

*Duddingtonia flagrans*, another nematode-trapping fungus, is the only species of its genus and is one of the main fungi studied as a biological control agent for gastrointestinal nematodes of production animals (Larsen 2006; Silva et al. 2013; Buzatti et al. 2015; Andrade et al. 2016). It has the capacity to produce conidia and many reproductive structures known as chlamydospores, a thicker light-brown spore type with higher resistance to environmental adversities (Rubner 1996). Chlamydospore cell walls are responsible for the high tolerance to gastrointestinal transit after oral administration, so *D. flagrans* can withstand digestive stress better than other species of nematophagous fungi (Assis et al. 2013), the source of the interest in its study. Once established in feces, infective parasitic larvae and free-living nematodes stimulate the production of adhesive hyphae in the form of traps that block and kill these nematodes, reducing their contamination on pastures (Braga and Araújo 2014).

Melanins are therefore enigmatic compounds that guarantee a variety of advantages to organisms, in addition to being associated with numerous defensive mechanisms against environmental damage (Eisenman and Casadevall 2012). Melanins are amorphous polymers that are difficult to study (Plonka and Grabacka 2006), so they have received much interest by the scientific community around the world. More detailed studies about their synthesis, structure, location within cells and role in the biology and survival of melanized fungi could provide a better understanding of how these macromolecules act so effectively in biological processes and survival resiliency. Chlamydospores have a thick cell wall that may be associated with tolerance to digestive stress, and melanin itself is present in the cell wall of various fungi, so we hypothesized that the survival of chlamydospores would be intimately correlated with the presence of melanin. The objective of our study was therefore to investigate the synthesis, distribution and function of this pigment in the ability of *D. flagrans* to survive ruminal stress by simulating the *in vitro* anaerobic incubation of the fungus with a rumen inoculum.

## Material and methods

### Conditions of cultivation and evaluation of glucose concentrations and ph

We identified the most suitable culture medium for obtaining fungal material by inoculating discs 10 mm in diameter of corn meal agar with a *D. flagrans* culture (CG 721) in YPD media (2% peptone, 1% yeast extract and 2% dextrose), PDA (0.4% potato extract, 2% dextrose and 1.5% agar), MM (minimal medium of 15 mM glucose, 10 mM MgSO_4_, 29.4 mM K_2_HPO_4_, 13 mM glycine and 3.0 mM thiamine, pH 5.5) and SDA (Sabouraud dextrose agar) supplemented with 1 mM of L-DOPA (Sigma-Aldrich, St. Louis, USA). The discs were incubated at 30°C for 21 days in a darkroom to avoid polymerization of L-DOPA. SDA medium was then chosen as the culture standard and was supplemented with various concentrations of glucose (2, 4, 8, 10 and 20% w/v) at pHs of 5.0, 5.5, 6.0, 6.5 and 7.0 for all formulations, also incubated at 30°C. Following this modification and with the fungus kept in a darkroom, 1 mM of L-DOPA was added to the medium to verify the increase in melanin pigmentation in the fungal strain.

### Influence of tricyclazole inhibitor on pigmentation

We evaluated the role of DHN in *D. flagrans* by preparing five Petri dishes (10 cm in diameter) containing SDA and tricyclazole (5-methyl-1,2,4-triazol[3,4] benzothiazole) (Sigma-Aldrich), an inhibitor of melanin biosynthesis. Ten milligrams of the inhibitor were dissolved in 1 mL of ethanol (solution stock) and added to the culture medium at concentrations of 20, 40, 60, 80, 160 and 320 μg/mL. SDA Petri dishes without the inhibitor were used as controls. The cultures were incubated at 30°C for 21 days in the dark.

### Obtainment of chlamydospores treated with L-DOPA, tricyclazole and a control without tricyclazole

To release the chlamydospores, each Petri dish containing *L-DOPA*, tricyclazole or a medium without tricyclazole was washed with 5 mL of 0.05% saline and 20% Tween with the aid of a scatter bar. The spores in solution were then carefully removed with a 100 μL micropipette, transferred to 10-mL centrifuge tubes and filtered through a 0.45 μm absorbent filter to obtain the chlamydospores.

### Isolation and purification of melanin

**Figure 1.**
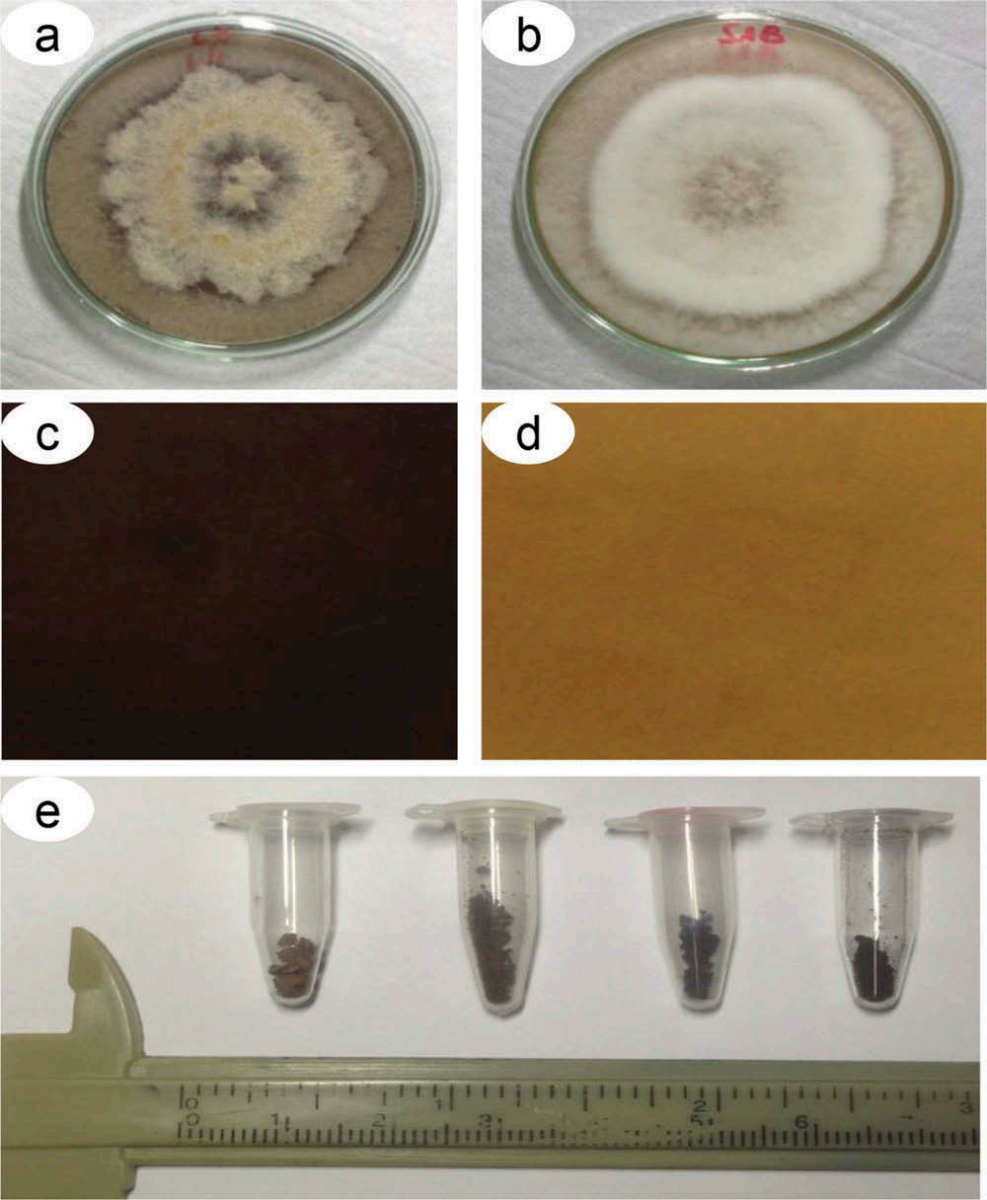
Pigmentation of *D. flagrans* colonies on Sabouraud dextrose agar. Colony view and culture medium with (a) and without (b) addition of L-DOPA and ventral view of a Petri dish with culture medium with (c) and without (d) addition of L-DOPA. e) Melanin particles extracted from five colonies grown in Petri dishes.

Melanin particles (Figure 1a) were extracted from the cultures of *D. flagrans* grown in SDA in the presence of 1 mM L-DOPA and 2% (w/v) glucose. The particles were isolated using denaturing agents and hot acid as described by Rosas et al. (2000) and Alviano et al. (2004): (i) batches of 21-d fungal colonies from five Petri dishes were centrifuged at 10 000 × g for 30 min, (ii) the pellet was washed in 0.1 M PBS, pH 7.5, (iii) the pellet was resuspended in 0.1 M sorbitol and 0.1 M sodium citrate, pH 5.5, (iv) this solution was centrifuged as previously described, *Trichoderma harzianum* lysis enzyme (1 mg/mL) was added and incubated at 30°C overnight for protoplast production, (v) protoplasts were collected by centrifugation as previously described and the pellet washed in PBS, (vi) 4.0 M guanidine thiocyanate was added and the solution was incubated at 25°C overnight, (vii) cell debris was collected by centrifugation as previously described, (viii) the pellet was washed three times with PBS, (ix) Proteinase K (1 mg/mL) and TRIS buffer were added and the solution was incubated at 37°C for 12 h, (x) the solution was centrifuged as previously described, and the pellet was washed three times with PBS, (xi) 6.0 M HCl was added and the solution was incubated at 65ºC for 1 h; (xii) the solution was centrifuged at 10 000 × g for 5 min, (xiii) the pellet was washed five times with PBS and (xiv) the solution was dialyzed with distilled water at room temperature for 10 d to obtain the polymers.

### Infrared spectroscopy (IS)

One milligram of the pigment was added to 100 mg of Potassium bromide (KBr) and formed into a tablet at 8 t cm^−2^ of pressure. One milligram of commercially purified synthetic melanin (Sigma-Aldrich) was used as a positive control. Infrared spectra were then obtained on an FT IR 8300 spectrophotometer (Shimadzu Corporation, Kyoto, Japan) in the region of 4000–400 cm^−1^.

### Electron paramagnetic resonance (EPR) spectroscopy

We used five samples for the EPR analyses: (i) 10 μg of the pigment isolated from *D. flagrans* as described above, (ii) fungal culture in Petri dishes with SDA medium supplemented with 1 mM L-DOPA, (iii) plates not supplemented with this precursor, which were used as controls, (iv) fungal culture in Petri dishes containing 160 μg of tricyclazole (Sigma-Aldrich) and (v) 1 mg of commercially purified synthetic melanin. The samples were crushed in a porcelain crucible and then spectrometrically analyzed. EPR spectra were obtained at room temperature (300 K) using an E500 spectrometer (Bruker Corporation, Billerica, USA) operating in X band (9.5 GHz), with microwave power of 1 mW, modulation amplitude of 1 G and modulation frequency of 100 kHz. A sample of MgO:Cr^3+^ (g of 1.9797) was used as a reference to obtain g for the signal of the sample.

### Transmission electron microscopy (TEM)

The samples were fixed at room temperature for 24 h in 2.5% glutaraldehyde (grade II) diluted with 0.1 M cacodylate buffer, pH 7.4. The samples were then washed three times for 15 min in the same buffer, and a subsample was post-fixed in 1% osmium tetroxide (OsO_5_) and 0.8% potassium ferricyanide for 60 min. Another subsample was washed in buffer, processed using Fontana-Masson technique adapted for TEM, as described by Franzen et al. (2008) and modified for the present study. The technique is based on a silver solution containing 10% silver nitrate in distilled water followed by the addition of ammonium hydroxide until a faint opalescence appears. The samples were then washed three times in distilled water and incubated in the ammoniated silver nitrate at 25°C for 40 min in a darkroom. The samples were then fixed again in glutaraldehyde diluted in 0.1 M cacodylate buffer as described above. The samples were again washed three times in distilled water and then dehydrated for 20 min in an increasing series of acetone (Merck, Darmstadt, Germany): 30, 50, 70, 90 and 100%. The same protocol of dehydration was followed for the samples fixed in 1% OsO_4_ and 0.8% potassium ferricyanide. The samples were then infiltrated slowly in an increasing series of acetone and Spurr resin (Sigma-Aldrich) starting at decreasing concentrations of acetone:Spurr of 3:1, 2:1, 1:1, 1:2, 1:3, until inclusion in pure resin. The inclusions were then conditioned in an oven at 60°C for 48 h for polymerization, and the blocks obtained were ultracut (Reichert Ultracut, Leica, Vienna, Austria). The ultrathin sections (70–90 nm) were collected onto copper (300 mesh) grids, contrasted in uranyl acetate and lead citrate for 20 and 5 min, respectively, dried and analyzed in a JEM-1400Plus transmission electron microscope (JEOL Ltd., Tokyo, Japan) at 80 kV.

### In vitro anaerobic stress with rumen inoculum

The use and maintenance of animals with permanent rumen cannulas was approved by the Committee on Ethics and Use of Animals (CEUA -UENF – protocol no. 380). *In vitro* incubations were carried out using 100-mL flasks containing reductive solution and culture medium prepared as described by Goering and Van Soest (1970) and containing ruminal inoculum prepared as described by Hall and Mertens (2008). The ruminal inoculum was obtained from cannulated steers kept on pasture and individually fed 1 kg/d of concentrate containing corn and soybean meal (3:1 on a dry matter basis). Individual samples of the liquid and solid contents of the rumen were collected, stored in thermal bottles and sent to the laboratory. Samples of ruminal contents were mixed in a blender for 60 s in a 1:2 ratio (solids:liquid) to detach bacteria and protozoa adhered to the solid digesta particles and to obtain a more representative sample of the ruminal microbiota. The mixture was then filtered through eight layers of gauze. The ruminal inoculum was added to the culture medium, previously reduced in a 4:1 ratio (reduced culture medium:rumen inoculum), and the mixture was maintained at 39°C under constant CO_2_ infusion until transference to the flasks, which were immediately sealed and kept in a water bath at 39°C (Hall and Mertens 2008). Approximately 0.5 g of tifton-85 (*Cynodon nlemfuensis* × *Cynodon dactylon*) sample was incubated with 48 mL of the culture medium containing the ruminal inoculum and 2 mL of the solution containing the chlamydospores for each treatment (L-DOPA, tricyclazole and a control without tricyclazole). The addition of tifton-85 prevented the depletion of nutrients from the culture medium. For each treatment we used three replicates and an aliquot of 5 mL from each flask collected at 0, 12, 24, 36 and 48 h of incubation to later analysis.

### Predatory activity of D. flagrans after in vitro incubation

Fecal samples were collected directly from the rectums of the 15 sheep to be used in fecal cultivation. The feces were thoroughly homogenized and submitted to the modified technique of Gordon and Whitlock (1939) for counting the number of eggs per gram (EPG) of feces. The positive fecal samples for nematode eggs were used in the predatory activity assays. For obtaining of infective larvae (L3), the coprocultures were made and based on the method described by Roberts and Sullivan (1950) with the following modifications: 4 g of feces were added to disposable 60-mL plastic cups. Chlamydospores from each treatment (L-DOPA, with and without tricyclazole) and time (0, 12, 24, 36 and 48 h) were individually inoculated in the cups at doses of 20 000 per mL, approximately. The cups were placed in a plastic box (30 cm long × 20 cm wide × 13 cm high) containing 100 mL of distilled water and covered with a plastic film to prevent loss of moisture during seven days of incubation at 27ºC. After incubation, each plastic cup was filled with distilled water and then capped with a Petri dish, and the cup was inverted. Ten milliliters of distilled water were added to the Petri dish. After 4 h, the distilled water was pipetted for recovery of L3 and for later quantification of these larvae. Each treatment and time had three replicates. A control group (without fungus) was also tested. The predatory activity was expressed in terms of survival as the rate:

(1) $$p_{ijk}=y_{ijk}/n_{ijk}$$

where $y_{ijk}$ is the number of surviving L3 from each Petri dish representing incubation time j, treatment i and replicate k. The total L3 count corresponds to $n_{ijk}$, and the number of dead larvae is $y_{ijk}-n_{ijk}$. The proportion $p_{ijk}$ represents the survival ratio. The distribution of $p_{ijk}∼Binomial\left(n_{ijk},\pi_{ijk}\right)$ and the GLIMMIX procedure (SAS Studio University Edition, SAS System Inc., Cary, USA) was used to quantitatively predict the survival based on the linear predictor and corresponding logit link function:

(2) $$\eta_{ijk}=\log\left(\pi_{ijk}/\left(1-\pi_{ijk}\right)\right)=\eta +\alpha_{i}+f_{j\left(i\right)}+\tau_{k}+\alpha \tau_{ik}$$

In which $\eta_{ijk}$ is the linear predictor; η is a constant; $\alpha_{i}$ is the effect of L-DOPA $\left(i=3\right)$, tricyclazole $\left(i=2\right)$ or a medium without tricyclazole as the control $\left(i=1\right)$; $\tau_{j}$ is the effect of incubation time; $\alpha \tau_{ij}$ is the interaction of both effects and $f_{j\left(i\right)}$ is the flask inoculated with rumen inoculum. The error was defined at the observational level, which was the Petri dish for each incubation time sample. A regression analysis was then performed by considering time as a continuous variable, because the interaction effect was considered significant$\left(P=0.001\right)$. The model was thus:

(3) $$\eta_{ijk}=\log\left(\pi_{ijk}/\left(1-\pi_{ijk}\right)\right)=\alpha_{i}+f_{j\left(i\right)}+\tau \alpha_{i}+\tau^{2}\alpha_{i}$$

In this case, an inverse-link function was needed to predict the survival of the nematodes and its 0.95 confidence intervals after fungal predation throughout the incubation times for each treatment on fungus growth. The inverse link was:

(4) $$E\left[\pi_{ijk}\right]=1/\left(1+\exp\left(-\left({\hat{\theta }}_{0i}+{\hat{\theta }}_{1i}\tau +{\hat{\theta }}_{2i}\tau^{2}\right)\right)\right)$$

In which $E\left[\pi_{ijk}\right]$ is the expected proportion of the population response, namely ${\hat{\pi }}_{ik}$, ${\hat{\theta }}_{0i}$ is the intercept for treatment i, ${\hat{\theta }}_{1i}$ is the linear coefficient for treatment i and ${\hat{\theta }}_{2i}$ is the quadratic coefficient of the linear predictor for treatment i. The linear and quadratic terms for continuous time are represented by τ and $\tau^{2}$, respectively.

## Results

### Effect of culture medium on pigmentation

The pigmentation of the colonies varied with the medium, time and supplementation used (Figure 1a-d). The colonies cultured in SDA were pigmented most when supplemented with 1 mM L-DOPA (Figure 1a, c) and incubated for 21 d. The colonies cultured without L-DOPA were not as dark as those supplemented with L-DOPA (Figure 1b, d). The addition of 2–8% (w/v) glucose at pH 5.5 was fundamental for melanization in *D. flagrans* (data not shown). The colonies cultured in YPD, PDA and MM did not develop well over time or did not melanize under the conditions evaluated.

### IS analysis

**Figure 2.**
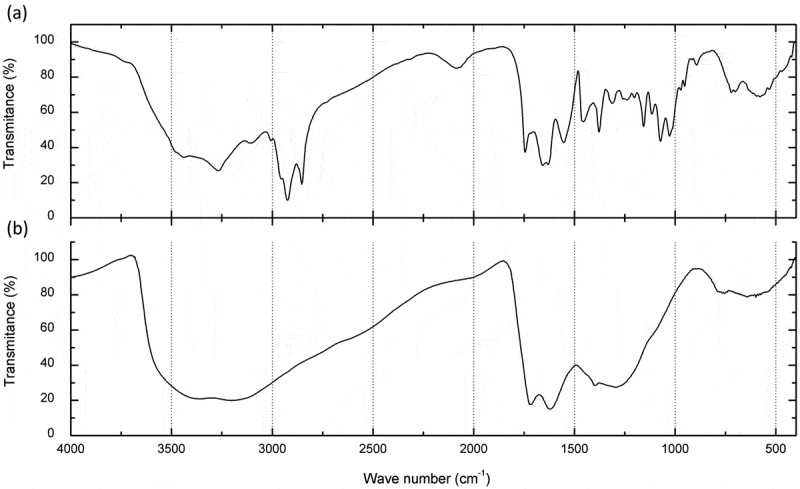
Infrared spectroscopic analysis. a) One milligram of melanin particles extracted from five colonies of *D. flagrans* and b) 1 mg of commercial melanin. Note that the amplitude of the signals demonstrates the similarity between the commercial and the *D. flagrans* melanin. The other wave spectra were due to the presence of radicals in the melanin particles extracted from the fungus.

The analyses demonstrated that the absorption bands for *D. flagrans* were consistent with the commercial synthetic melanin used as a control (Figure 2a, b). Based on the results for the spacing regions of the melanin polymers extracted from *D. flagrans*, we could identify the functional groups responsible for spectrum absorption: hydroxyls of associated strong bands and chelates (H^+^, C = O and NO_2_) in the regions between 3441 and 2852 cm^−1^; N = C = S (intense and large bands of isethionate), C = O of esters, associated amides and non-substituted amides; C = C of aromatic chain; NH_2_ of strong media bands and CH_2_ and CH_3_ in the corresponding region between 2087 and 1377 cm^−1^; C-F (halogen), C-O of carboxylic acids, unsaturated esters and aromatic chain and CO of aromatic-aliphatic esters and vinyl groups in the region between 1311 and 1072 cm^−1^; S = O (sulfoxide), R-CH = CH_2_, -C = CH_2_, R_2_-C = CH_2_, -CH = CH_2_ and various aromatic rings in the region between 1028 and 702 cm^−1^; chloroalkanes between 596 and 563 cm^−1^ and compounds formed by bromoalkanes and iodine alkane in the corresponding regions between 532 and 470 cm^−1^.

### EPR spectroscopy

**Figure 3.**
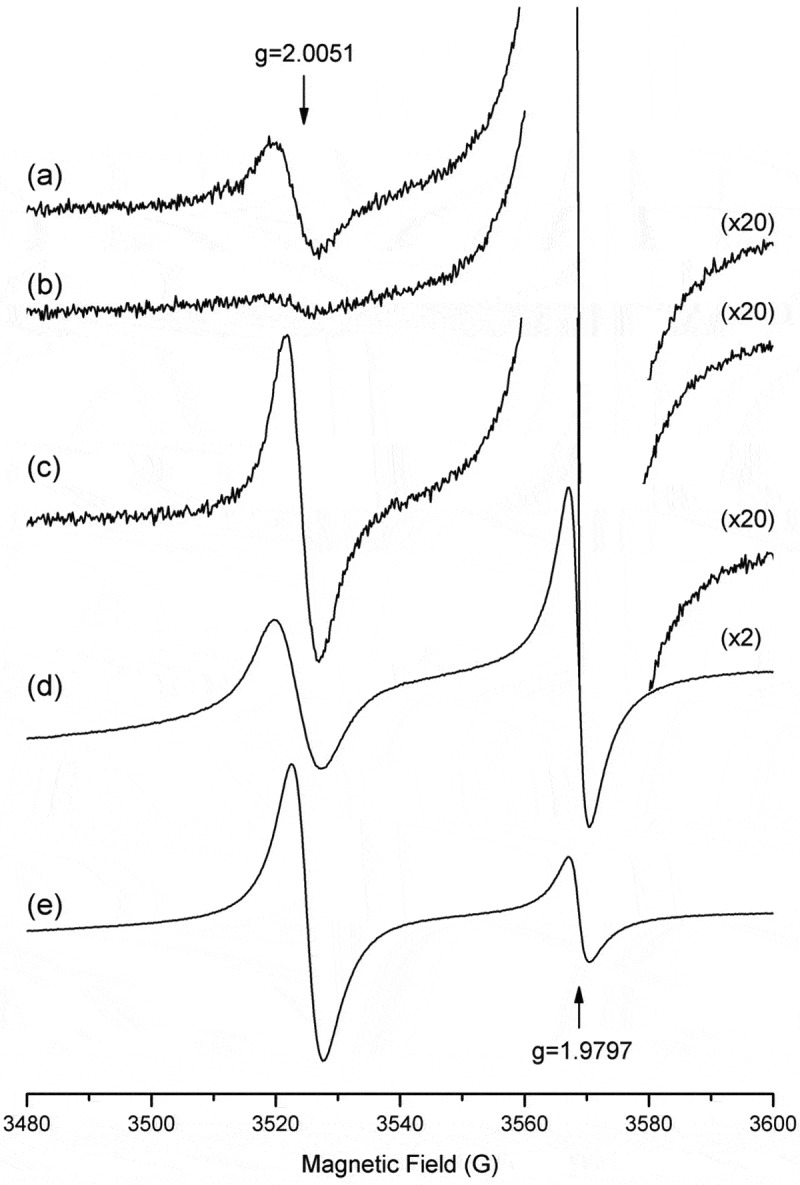
EPR spectra. a) Colony of *D. flagrans* in SDA medium, b) *D. flagrans* treated with 160 μg of tricyclazole, c) *D. flagrans* colony supplemented with 1 mM L-DOPA, (d) melanin particles extracted from *D. flagrans* and (e) commercial melanin. The 84% lower free-radical concentration can be seen in the tricyclazole-treated sample (b), and an increase of up to three-fold can be seen in the sample supplemented with L-DOPA(c), relative to no treatment (a). A sample MgO:Cr3^+^ (g of 1.9797) was used as a reference to obtain g for the sample studied.

The spectra of the commercial melanin and the fungal melanin from cultures of *D. flagrans* supplemented with 1 mM L-DOPA and 160 μg of tricyclazole had an isotropic line with a g of 2.0051 ± 0.0001, indicating that the same free radical was detected in all samples. The free-radical concentration was 84% lower in the treatment with 160 μg tricyclazole (Figure 3b) than the fungal culture without this treatment (Figure 3a). The concentration of the free radical was three-fold higher in the sample supplemented with L-DOPA (Figure 3c) than the sample without any treatment (Figure 3a). The melanin extracted from colonies of *D. flagrans* (Figure 3d) and the commercial melanin (Figure 3e) had free-radical concentrations identical to that of untreated fungus (Figure 3a).

### Inhibition of melanization by tricyclazole

**Figure 4.**
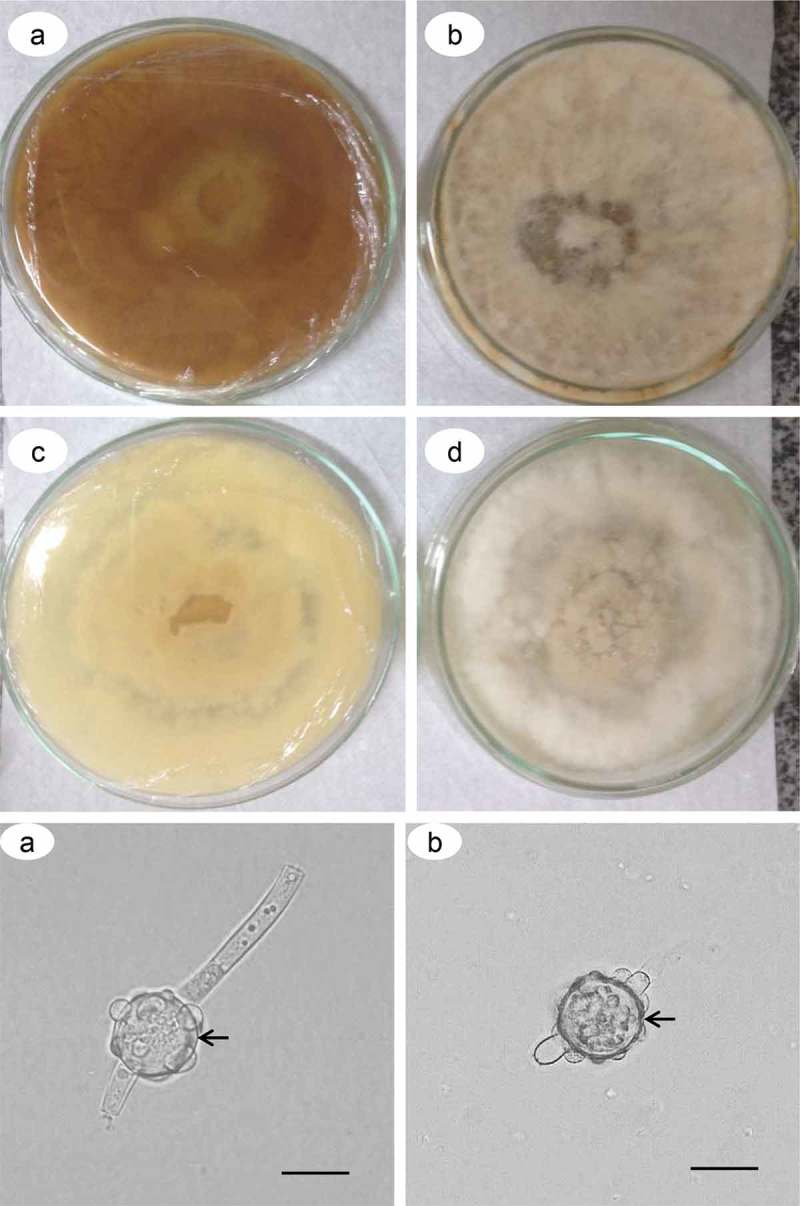
Inhibition of melanization by tricyclazole in the *D. flagrans* colonies. Colony view and culture medium without (a, b) and with (c, d) treatment with 160 μg/mL of tricyclazole. Chamydospores from plates with (e) and without (f) treatment with 160 μg/mL of tricyclazole. Cell wall (arrow). Bars: 50 μm.

All colonies treated with 20, 40, 60, 80, 160 and 320 μg of tricyclazole did not develop pigmentation as dark as those not treated and supplemented with L-DOPA (Figure 4a – d), but the EPR analysis of the sample treated with 160 μg of tricyclazole indicated the presence of the free radical characteristic of melanin. Samples cultured with 320 μg of tricyclazole had deficient development. Chlamydospores and conidia were produced, but in smaller quantities, and were more transparent. The alteration in pigmentation on the chlamydospores was perceptible, as shown in the 160 μg tricyclazole treatment by optical microscopy (Figure 4e – f).

### TEM structural analysis

**Figure 5.**
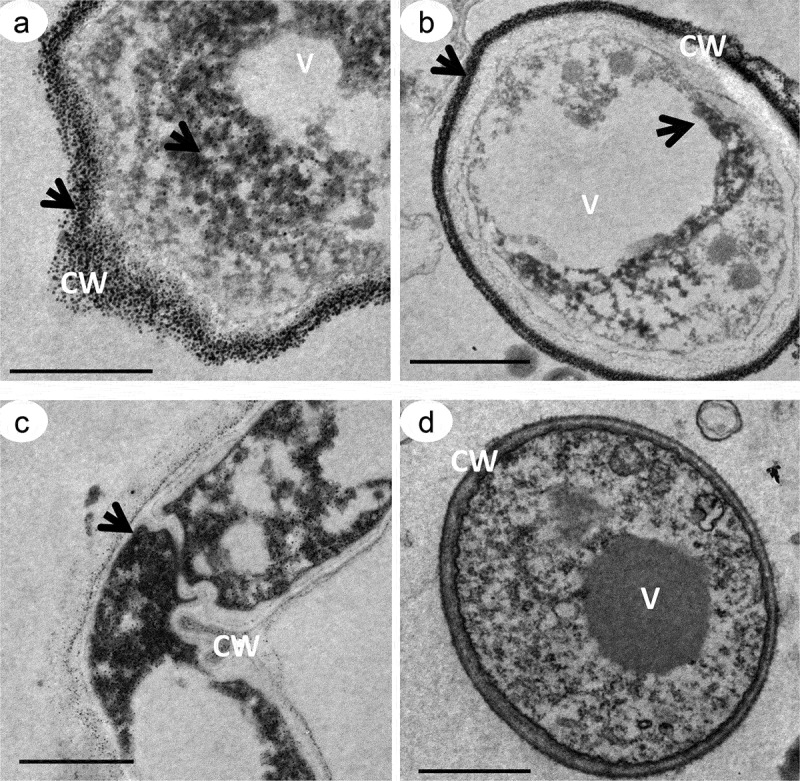
Transmission electron microscopy of *D. flagrans* demonstrating the location of the electron-dense particles of melanin. Culture medium with addition of L-DOPA. Fungal hypha (a, c), chlamydospores (b, d), cell wall (CW) and vacuoles (V). Samples contrasted with 10% silver nitrate (a, b), and samples contrasted with 1% osmium tetroxide and 0.8% potassium ferricyanide (c, d). The arrows indicate the electron-dense particles. Scale bars: 5 μm (a-c) and 2 μm (d).

**Figure 6.**
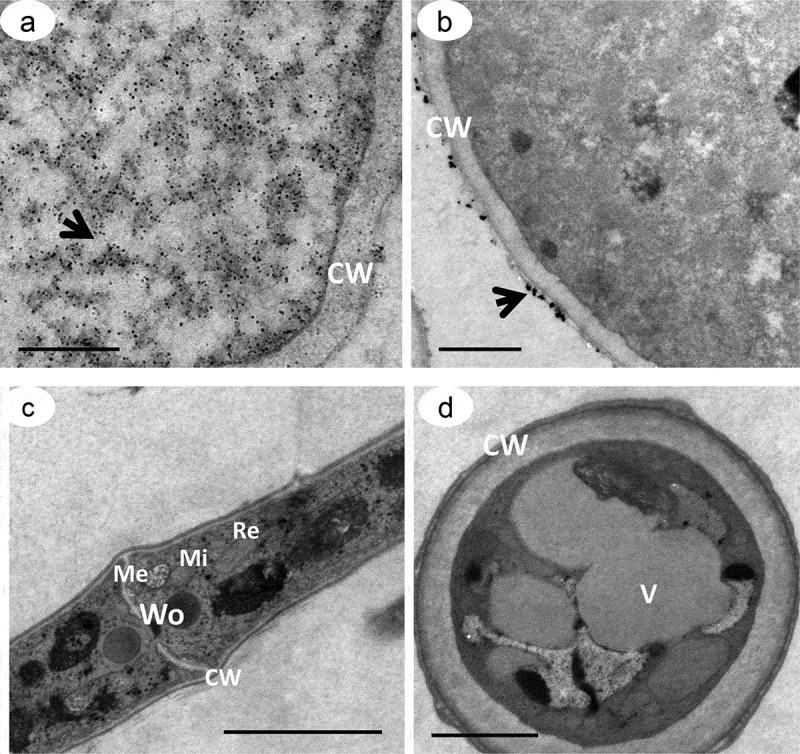
Transmission electron microscopy of *D. flagrans* treated with 160 μg/mL of tricyclazole, an inhibitor of melanin synthesis. Culture medium without addition of L-DOPA. Note the reduced distribution of the electron-dense particles throughout the cell (a-d) compared to Figure 5. The arrows indicate the melanin particles. Note the absence of melanin granules for the samples stained with 1% osmium tetroxide and 0.8% potassium ferricyanide (c, d). Cell wall (CW), melanosome (Me), mitochondria (Mi), vacuoles (V), endoplasmic reticulum (Re) and Woronin body (Wo). Scale bars: 5 μm (a) and 2 μm (b-d).

**Figure 7.**
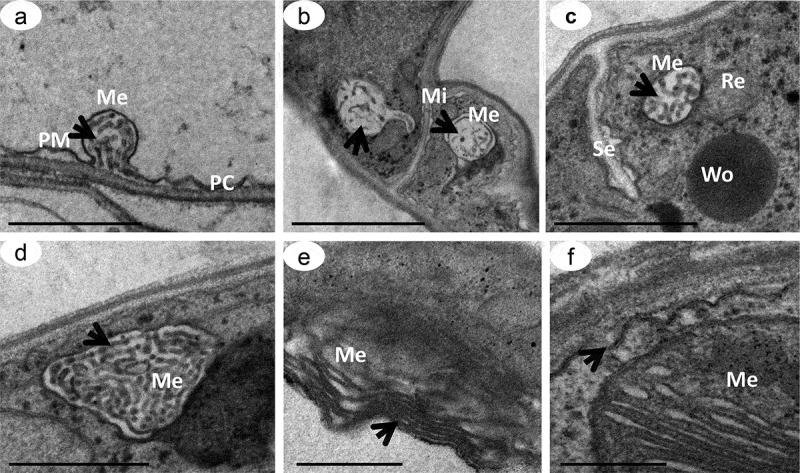
Transmission electron microscopy of *D. flagrans* melanosomes. Ultrathin slices demonstrating the formation of melanosomes in stages II to III (a-d) in cells treated with 160μg/mL of tricyclazole and in stage III in cells treated with 1mM L-DOPA (e-f). Melanosome (Me), plasma membrane (PM), mitochondria (Mi), Woronin body (Wo) and septum between hyphae (Se). Arrows show the arrangements of internal matrices in the melanosomes. Scale bars: 500 nm (a, d, e, f) and 1 μm (b, c).

The ultrastructure of hyphae and chlamydospores stained with 10% silver nitrate indicated the presence of electron-dense particles distributed throughout the cytoplasm, with greater deposition on the cell-wall surface (Figure 5a, b). The same pattern of pigmentation, but less intense, was observed for samples stained with osmium tetroxide and potassium ferricyanide (Figure 5c, d). The opposite was observed for samples treated with 160 μg of tricyclazole, which had fewer electron-dense particles distributed in the cytoplasm and on the cell wall, even when labeled with the ammoniacal silver-nitrate solution (Figure 6a-d). The ultrathin sections of *D. flagrans* also indicated a subcellular compartment with a fibrillar structure and different profiles of deposition of electron-dense particles identified as a melanosome (Figure 7a-f).

### Predatory activity of D. flagrans post ruminal stress

**Figure 8.**
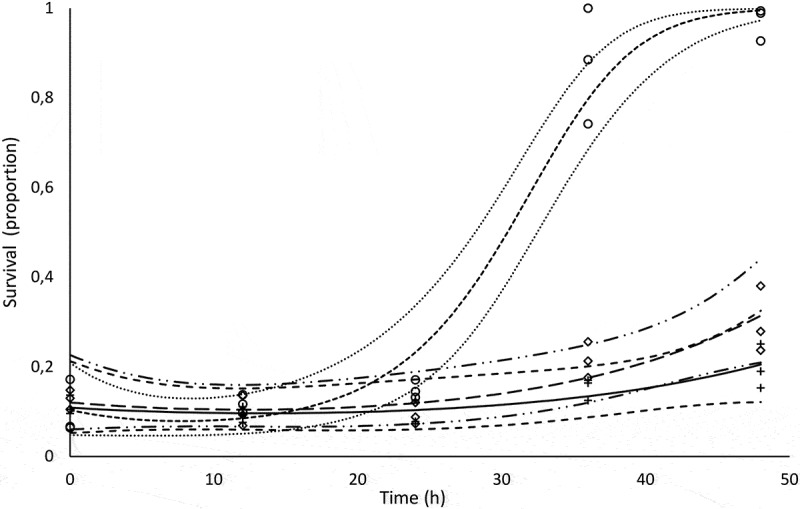
Observed and predicted survival proportions of the L3 larvae inoculated with chlamydospores of *Duddingtonia flagrans*. Diamonds, circles, and crosses are observed survivals for L-DOPA, tricyclazole and control, respectively. The small-spaced dashed line is the predicted survival of L3 with fungal melanin inhibitor (tricyclazole), and the dotted lines above and below it are 0.95 confidence limits (0.95CI). The solid line is the predicted L3 survival in the control medium with the fungus, and the large-spaced dashed lines form the 0.95CI. The long-dashed line is the predicted survival of L3 with L-DOPA melanin stimulant, and the dashed-double-dotted lines correspond to 0.95CI.

The interaction between anaerobic incubation with ruminal inoculum and the treatments of growing medium with L-DOPA, medium with tricyclazole and medium without tricyclazole, as previously mentioned, was significant$\left(P=0.001\right)$. A quadratic regression effect in the linear predictor with this interacting effect sliced per treatment was therefore fitted, and the survival behavior was predicted (point and 0.95 confidence-interval estimates) and presented as natural proportions (Figure 8). After considering the 0.95-interval estimates, the L3 survival tendencies were disjoint higher for the tricyclazole after 27 h of anaerobic incubation with rumen inoculum, indicating an important and significant reduction in the predation of the larvae by the fungus *D. flagrans* that grew in the presence of this melanin inhibitor. The predatory capacity of the fungus that grew over control and L-DOPA growth media over L3 larvae, within the limits of statistical error, remained active and unaltered.

## Discussion

Melanins are heteropolymeric, negatively charged, high-molecular-weight, amorphous substances with a variety of functions, are distributed throughout the biological kingdoms (Eisenman and Casadevall 2012). Structurally, melanins are a group of complex pigments and are often provisionally classified based on their color (Treseder and Lennon 2015). We thus analyzed the influence of culture medium on pigmentation in *D. flagrans* supplemented with 1 mM L-DOPA, using glucose as a carbon source at concentrations of 2–20% (w/v). The hyphae were pigmented only in the presence of L-DOPA, which is a key component for the production of melanin, as previously demonstrated for *Trichosporon asahii, T. asteroides, T. inkin* and *T. mucoides* (MHG et al. 2014).

*D. flagrans* pigmentation was higher at glucose concentrations similar to the commercially available Sabouraud culture media, which was added to the medium at 2% and intensified to maximal staining at concentrations of 2–8%. Almeida-Paes et al. (2009) reported similar results, where melanization in *Sporothrix schenckii* was restrictive in media without glucose and much higher in media containing up to 10% glucose for some strains and L-DOPA, which was fundamental for melanogenesis. Pigmentation occurred later for SDA medium than the other media, and the higher glucose concentration did not prevent pigmentation in *S. schenckii*. These results support those of the present study, where cultures of *D. flagrans* became pigmented when incubated at 30°C for 21 d with concentrations of up to 8% glucose. MHG et al. (2014) also observed better melanization in *Trichosporon* spp. in the presence of high concentrations of glucose and L-DOPA and suggested that melanization could be controlled by nutritional factors. Frases et al. (2006) also reported similar results for cultures of *Cryptococcus neoformans*, in which dark staining, typical for melanin, was totally dependent on the medium used, but dark pigmentation was not observed for SDA medium. We also observed that glucose concentrations >10% inhibited the development of colonies of *D. flagrans*.

The role of DHN in fungi has been extensively studied with the use of inhibitors of melanin biosynthesis. The Melanosomes, intracellular organelles with the exclusive function of synthesizing and to stock melanin (Eisenman and Casadevall 2012) pass through four stages morphologically distinct (Slominski et al. 2004), being the first and second stages, characterized by the complete absence of melanin and the formation of intralamellar fibers within its structure (Raposo and Marks 2007). Thus, the addition of tricyclazole affects the inhibition of THN reductase and acts on scytalone, thereby reducing the concentration of intermediate metabolites during melanin synthesis, which in turn can self-oxidize and accumulate 2-hydroxyjuglone (2-HJ) and flaviolin (Romero-Martinez et al. 2000) immediately after the end of intralamellar fiber formation, which in fungi, leads to a depletion in the synthesis, the carriage and the deposition of pigment in the cell walls. However, not affecting the construction of the organelle. The 84% lower free-radical concentration in the tricyclazole-treated culture suggests that this free radical is associated with the presence of melanin. Several studies have reported that tricyclazole can significantly decrease mycelial staining in various fungi widely recognized as producing melanin (Lazarovits and Stoessl 1988; Howard and Valent 1996; Rižner and Wheeler 2003; Zhang et al. 2006; Kumar et al. 2015), but does not completely inhibit staining, even at higher inhibitor concentrations (Mares et al. 2004). An increase in the amount of tricyclazole to 160 μg decreased the growth of *D. flagrans* considerably but did not completely inhibit melanin production, based on the EPR spectrum in the tricyclazole-treated sample.

Melanin synthesis involves reduction and/or oxidation reactions followed by polymerization of phenolic and indolic compounds leading to the final organic polymers (Slominski et al. 2004). The intermediate free radicals formed within this complex provide melanin with paramagnetic properties, which may facilitate EPR spectroscopy for detecting and safely investigating the presence of free radicals in melanin samples (Meredith and Sarna 2006). We obtained an EPR resonance signal that confirmed the presence of a free radical with a g of 2.0051 ± 0.0001 for all samples (commercial sample, sample of melanin particles extracted from the fungus cultured in SDA, fungal samples supplemented with L-DOPA and samples treated with 160 μg of tricyclazole). This radical may have been *o*-semiquinone, an endogenous free radical characteristic of melanin (Meredith and Sarna 2006). Similar signals were also observed by Cesareo et al. (2012) in a study of the use of EPR for diagnosing human melanoma cells and by Otręba et al. (2015) in a study with human melanocytes (g of 2.0021 for both). The free radical with a g of 2.0051 detected in our samples would therefore correspond to a semiquinone and could be associated with the production of melanin by *D. flagrans*.

The morphological and ultrastructural study of *D. flagrans* cells identified melanin granules in cohesion with the cell wall, either as polymers incorporated within the wall or in its outermost layer. Mandal et al. (2007) similarly demonstrated the presence of melanin on the cell wall of *C. neoformans* in the form of concentric rings of different sizes and occupying approximately 75% of the cell-wall area compared to albino strains. Zhong et al. (2008) detected covalent bonds between polysaccharides and nanoproteins in intimate association with melanin in the cell walls of *C. neoformans* and suggested that melanin could act as a component of the cell wall. Franzen et al. (2006) also verified the presence of melanin granules deposited on the cell wall of the mycelium and the conidia of *Fonsecaea pedrosoi*.

The position of the melanin layer is an important factor in determining the function of this polymer in protecting against damage from the surrounding environment, and the dense network of multiple layers of melanin on the cell wall of the chlamydospores in *D. flagrans* suggest how a resistance structure, which has been widely studied for the biological control of gastrointestinal nematodes in production animals, could restrict the passage of nematodes through the digestive tract of these animals long before their final action in the capture and predation of nematodes in the stool. Eisenman et al. (2005) found that melanin in *C. neoformans* was distributed over several layers of granules that formed small pores and channels between the particles; specific anti-melanin antibodies reduced the size of these pores relative to control cells, so small molecules could be prevented from entering the cell through its wall.

We demonstrated the presence of highly specialized subcellular structures similar to the mammalian lysosomes known as melanosomes (Seiji et al. 1963) that appear to participate directly in the synthesis and deposition of melanin in *D. flagrans*. Franzen et al. (2008) reported that melanin synthesis by *F. pedrosoi* began inside the melanosomes with the formation of a fibrillar matrix followed by junction with the fungal membrane, from which the melanin granules could reach the cell wall, occupying the periplasmic spaces between the membrane and the wall of the fungus. The images obtained, however, did not clearly illustrate the similarity of the mentioned descriptive processes, as with the melanosomes of animal cells.

Melanosomes in mammals and at least one species of salamander (Prelovšek and Bulog 2003) have four stages of maturation: stage I – pre-melanosome, with a filamentous matrix; stage II – the lamellar filaments are well defined and tyrosinase activity is high; stage III- beginning with the deposition of the melanin granules and high enzymatic activity along the lamellar matrix and stage IV – the complete deposition of melanin and increased opacity and darkening of the organelle (Orlow 1995; Raposo et al. 2001; Raposo and Marks 2007; Miot et al. 2009). Similar maturation stages of melanosomes have been proposed for fungi but had not yet been well documented, as we have for *D. flagrans*.

It is important to note that the arrangement of melanin on the cell wall in fungi tends to differ between species, but its presence has been well described for several species as an important factor of protection and virulence, essential for survival mainly in free-living species (Eisenman and Casadevall 2012). Fernandez and Koide (2013) observed that melanin in hyphal cell walls of the ectomycorrhizal fungus *Cenococcum geophilum* was an important regulator of tolerance to osmotic stress and desiccation in environments subject to water stress. Melanized cell walls can thus help to prevent the release of solutes from the cell, increasing the success of the fungus under possible osmotic stress, and melanization could strengthen the cell wall, allowing the cell to support the high turgor pressure resulting from next to water stress. The high survival of digestive stress by *D. flagrans* has been attributed to the chlamydospores and especially to their thick cell walls (Ojeda-Robertos et al. 2009). We, however, demonstrated a thick deposit of melanin on the cell wall, which may be associated with the resistance to digestive stress by decreasing wall porosity, because melanization can interfere with porosity by decreasing the size of cells (Jacobson and Ikeda 2005).

The *in vitro* ruminal incubation in the present study interfered in the predatory capacity of the fungus treated with tricyclazole. Predation remained active in the control and L-DOPA treatments, suggesting that the absence of melanin in the cell wall of these chlamydospores induced by tricyclazole treatment rendered them susceptible to the simulated *in vitro* anaerobic conditions of the ruminal environment.

Our results indicated that *D. flagrans* synthesized melanin. The melanin particles were structurally associated with the cell wall, suggesting that melanin may act by protecting chlamydospores against ruminal stress. Our findings are the first record for nematophagous fungi indicating the presence of melanosomes in the synthesis and deposition of melanin.
